# Fluctuations in Intestinal Microbiota Following Ingestion of Natto Powder Containing *Bacillus subtilis var. natto* SONOMONO Spores: Considerations Using a Large-Scale Intestinal Microflora Database

**DOI:** 10.3390/nu14183839

**Published:** 2022-09-16

**Authors:** Kanako Kono, Yasufumi Murakami, Aya Ebara, Kana Okuma, Hidetaka Tokuno, Ayano Odachi, Kazuya Ogasawara, Emi Hidaka, Teruaki Mori, Kazuko Satoh, Shingen Kimoto, Hiroaki Masuyama, Midori Takeda, Shunsuke Managi

**Affiliations:** 1 Symbiosis Solutions Inc., Tokyo 101-0064, Japan; 2 Department of Biological Science and Technology, Tokyo University of Science, Tokyo 162-8601, Japan; 3 Sonomono Inc., Fukuoka 810-0023, Japan; 4 Department of Agricultural and Resource Economics, Faculty of Agriculture, Kyushu University, Fukuoka 819-0395, Japan; 5 National Hospital Organization Nishi-Beppu National Hospital, Oita 874-0840, Japan; 6 Department of Nursing, Faculty of Medicine, Oita University, Oita 879-5593, Japan; 7 Kimoto and Mori Medical Holdings, Oita 875-0001, Japan; 8 Urban Institute & Department of Civil Engineering, Kyushu University, Fukuoka 819-0395, Japan

**Keywords:** intestinal microbiota, gut microbiota, 16S rRNA, *Bifidobacterium*, Blautia, *Bacillus subtilis var. natto* SONOMONO, fermentation, natto

## Abstract

Improving the intestinal microbiota using probiotics, prebiotics, and synbiotics has attracted attention as a method of disease prevention and treatment. This is the first study to discuss the effects of food intake on the intestinal microbiota using a large Japanese intestinal microbiota database. Here, as a case study, we determined changes in the intestinal microbiota caused by ingestion of a processed natto food containing *B. subtilis* *var. natto* SONOMONO spores, SONOMONO NATTO POWDER CAPSULES^TM^, by analyzing 16S rRNA sequence data generated using next-generation sequencing techniques. The results showed that the relative abundance of *Bifidobacterium* and *Blautia* as well as the relative abundance of *Bifidobacterium* were increased in males and females in the ingesting group, respectively. Additionally, the effects of SONOMONO NATTO POWDER CAPSULES^TM^ intake on *Bifidobacterium* and *Blautia* abundance depended on the relative abundance of *Bifidobacterium* at baseline. Finally, analysis of a large Japanese intestinal microbiota database suggested that the bacterial genera that fluctuated with the ingestion of SONOMONO NATTO POWDER CAPSULES^TM^ may be associated with lifestyle-related diseases such as diabetes.

## 1. Introduction

Research on the human intestinal microbiota has seen great advances, with improvements in bacterial isolation, culture techniques, and phylogenetic classification [1,2,3]. In recent years, 16S rRNA metagenomic analysis has been used to analyze the intestinal microbiota of patients with various diseases and to characterize the intestinal microbiota of healthy individuals. As a result, it has become clear that the diversity of the intestinal microbiota and abnormal bacterial species composition (dysbiosis) are involved in various diseases [4,5,6]. In addition, as 16S rRNA metagenomic analysis has identified multiple changes in the intestinal microbiota following food intake, improvement of intestinal microbiota using probiotics (microorganisms that have beneficial effects on the host), prebiotics (foods that selectively alter the growth and activity of specific bacteria in the colon and have beneficial effects on the host), and synbiotics (a combination of probiotics and prebiotics) is attracting attention as a method of disease prevention and treatment [7,8,9]. Furthermore, the characteristics of the intestinal microbiota are influenced by factors such as age, sex, and geographical location, and it has been reported that the characteristics of the intestinal microbiota in healthy people differ between countries [10,11]. Therefore, it is significant, as it enabled us to determine the effects of changes in the intestinal microbiota due to food intake on the health status of people by comparing them with large-scale data from people living in the same area.

Natto is a traditional Japanese fermented food made by fermenting steamed soybeans with *Bacillus subtilis var. natto*. The fermentation process produces a variety of functional molecules [12]. Natto is rich in soy-derived proteins and vitamin K, which is essential for the production of blood coagulation factors. Natto is also rich in dietary fiber, which is thought to have a beneficial effect on intestinal regulation. In particular, natto is expected to be an excellent probiotic because it contains *B. subtilis var. natto* itself [13]. A study investigating the effects of natto on the gut microbiota reported an increase in *Bifidobacterium* abundance following natto consumption [14]. There are also reports that natto intake can prevent diabetes [15] and dyslipidemia [16] by suppressing the increase in postprandial blood glucose levels.

This is the first study to make use of the large Japanese gut microbiota database containing 16S rRNA metagenomic data to discuss the effects of food intake on intestinal microbiota. In this case study, 16S rRNA metagenomic analysis was used to analyze the effects of ingesting natto processed food for 62 days on the intestinal microbiota. Since sex differences affect the intestinal microbiota, analyses were conducted separately for males and females.

## 2. Materials and Methods

### 2.1. Study Population

A total of 205 Kohoku residents in Saga Prefecture, Japan (100 males and 105 females), aged 20 to 89 years, who fully understood the purpose, maintenance, usage, and safety of the research, gave their consent for participation in the study. All participants gave their informed consent for inclusion before they participated in the study. The study was conducted in accordance with the Declaration of Helsinki, and the protocol was approved by the Ethics Review Board of the sonomono Inc., Fukuoka, Japan (protocol no. 2020001; 2 October 2020). Those who met the following exclusion criteria were excluded from the study: (1) had a history of abnormal laboratory values or cardiopulmonary function and were judged to have problems participating in the study, (2) were at risk of developing a serious allergy associated with the study product, (3) had a disease requiring constant medication or a history of a serious disease requiring treatment, (4) were participating in other clinical trials at the start of this study, (5) were planning to become pregnant or were breastfeeding during the study period, and (6) were deemed inappropriate by the investigators.

Kohoku Town is located in the central part of Saga Prefecture, Japan. Its main industries are agriculture and livestock farming. Medical expenses associated with diabetes and dyslipidemia are increasing in Kohoku Town, and according to health checkup data, the percentage of Kohoku Town residents with a HbA1c level of 5.6% or higher (subject to guidance during metabolic syndrome health checkups) is above the national average for both males and females.

### 2.2. Study Design

The study was an open study in which participants of both sexes were randomly divided into two groups: one group ingested the test product (LNC group: Lyophilized natto capsules group), and the second group did not ingest the test product (control group). Participants assigned to the LNC group were asked to ingest three SONOMONO NATTO POWDER CAPSULES^TM^ per day for 62 days (from November 2020 to January 2021).

Stool samples from individuals in the LNC group were collected at three time points: before intake (Day 0), 31 days after intake (Day 31), and 62 days after intake (Day 62). Stool samples from participants in the control group were collected at the same time points. Stool samples were collected by the participants themselves. Additionally, the participant’s background information was investigated using a self-reporting system. The background information collected included age, sex, height, weight, lifestyle, health status, and dietary habits. During the intake period, participants were asked to fill a checklist every day, including whether they ingested the test product, their body weight, whether and how often they defecated, the condition of their stool (quantity, shape, color, and odor), their physical condition, whether they took any drugs, and whether they ate other fermented foods, lactic acid bacteria beverages, or dietary supplements. They were instructed not to change their usual dietary habits during the study period.

### 2.3. Test Product

SONOMONO NATTO POWDER CAPSULES^TM^ contain 380 mg of lyophilized natto powder made from 100% “Fukuyutaka” soybeans—grown in Kohoku Town without chemical pesticides or fertilizers—in a hard-shell capsule made of plant-derived hydroxypropyl methylcellulose (HPMC). Each gram of lyophilized natto powder contains 1.6 × 10^10^ colony forming units (CFUs) of Bacillus natto in the spore state. *Bacillus subtilis var. natto* SONOMONO was used as the Bacillus natto strain. The strain was selected by single colony isolation from commercially available natto strains and is characterized by lower production of odor and stickiness components and higher protease activity compared with common natto strains (unpublished data). The intake was three capsules per day (1140 mg of natto powder and 1.8 × 10^10^ CFUs of *Bacillus subtilis var. natto* SONOMONO).

### 2.4. Stool Sample Collection

Stool samples were collected by the participants using a stool collection kit (TechnoSuruga Laboratory, Co., Ltd., Shizuoka, Japan), suspended in guanidine thiocyanate (GTC) solution (100 mM Tris-HCL (pH 9.0), 40 mM Tris-EDTA (pH 8.0), 4 M guanidine thiocyanate, and 0.001% bromothymol blue and mailed at room temperature.

### 2.5. DNA Extraction

DNA was extracted from stool samples using a fully automated enterobacterial DNA extractor (DEX-I; PMT Corporation, Fukuoka, Japan). Briefly, 100 µL of lysozyme (10 mg/mL) and 400 mg of glass beads were added to 900 µL of GTC solution containing a stool sample the size of a grain of rice in suspension, stirred, and allowed to stand at 50 °C for 30 min. Then, 500 µL of TE-saturated phenol-chloroform-isoamyl alcohol solution (25:24:1) and 110 µL of 10% SDS solution were added to the sample. Samples were processed at 2500 rpm for 2 min in a bead crusher (PMT Corporation, Fukuoka, Japan) and then incubated at 70 °C for 10 min. Sample crushing and incubation were performed twice. The samples were cooled, centrifuged at 13,200× *g* for 10 min, and the supernatant aliquoted. Next, 700 µL of isopropyl alcohol and 70 µL of 3 M sodium acetate solution were added to the supernatant, the solution stirred and centrifuged at 13,200× *g* for 15 min, and the supernatant discarded. The precipitated DNA pellet was washed twice with 70% ethanol solution, dissolved in 200 µL of TE buffer, and then stored at −80 °C until DNA sequencing.

### 2.6. DNA Sequencing

Using 12.5 ng of DNA extracted from stool specimens, variable regions V1 to V3 of the 16S rRNA gene was amplified using 35F primer (5′-TCGTCGGCAGCGTCAGATGTGTATAAGAGACAGCCTGGCTCAGGATGAACG-3′) [17] and 520R primer (5′-GTCTCGTGGGCTCGGAGATGTGTATAAGAGACAGACCGCGGCTGCTGGC-3′). PCR amplifications were carried out in 25 µL solutions containing 0.2 mM dNTPs, 0.2 µM of each primer, 0.5 U Q5 Hot Start High-Fidelity DNA Polymerase (New England Biolabs, Ipswich, EX, USA), 1 × Q5 Reaction Buffer, and 12.5 ng of sample DNA. The following thermal cycling conditions were used: initial denaturation at 98 °C for 3 min followed by 20 cycles of denaturation (98 °C for 10 s), annealing (55 °C for 30 s), and extension (72 °C for 1 min), and a final extension at 72 °C for 7 min. PCR products were purified with 20 µL of AMPure XP (Beckman Coulter, Inc., Brea, CA, USA) and eluted into 50 µL of 10 mM Tris-HCl, pH 8.5.

To prepare a DNA library for Illumina MiSeq sequencing using Nextera XT Index Kit v2 primers (Illumina, San Diego, CA, USA), PCR reactions were performed in 50 µL solutions containing 0.2 mM dNTPs, 5 µL of each primer, 1 U Q5 Hot Start High-Fidelity DNA Polymerase, 1 × Q5 Reaction Buffer, and 5 µL of the eluted DNA. The following thermal cycling conditions were used: initial denaturation at 98 °C for 3 min followed by 8 cycles of denaturation (98 °C for 10 s), annealing (55 °C for 30 s), and extension (72 °C for 1 min), and a final extension at 72 °C for 7 min. PCR products were purified with 56 µL of Agencourt AMPure XP in accordance with the manufacturer’s protocol and eluted into 25 µL of 10 mM Tris-HCl, pH 8.5. The concentration of the index PCR product was measured using the QuantiFluor (R) dsDNA System (Promega, Madison, WI, USA) and then diluted to 4 nM. Five microliters of each sample was used to generate a sequencing library and denatured in 0.2 N sodium hydroxide solution to a final concentration of 8 pM. To this solution, 8 pM of denatured PhiX was added to obtain a final concentration of 25–40% (*v*/*v*). The adjusted solution was processed on a MiSeq system (Illumina, San Diego, CA, USA) using Reagent Kit v3 (Illumina, San Diego, CA, USA) for DNA sequencing. The DNA sequencing process consisted of two rounds of 300 cycle reactions.

### 2.7. 16S rRNA Data Analysis

Fastq files were created from the base call (bcl) MiSeq file outputs using the bcl2fastq software ver. 2.20.0.422 (Illumina, San Diego, CA, USA). The generated fastq files were processed using clsplitseq ver. 0.2.2019.05.10 (https://www.claident.org/, accessed on 1 January 2022) to remove primer sequences and create demultiplexed fastq files. The quality score in clsplitseq was set to 20. The generated overlapping and paired-end fastq files were processed using DADA2 ver.1.16 package in the R software ver. 4.0.3 (R Foundation for Statistical Computing, Vienna, Austria) to create amplicon sequence variants (ASVs) [18]. The DADA2 package was executed using the DADA2 pipeline version 1.16 (https://benjjneb.github.io/dada2/tutorial.html, accessed on 1 January 2022). The filterAndTrim function arguments used were truncLen = c(0, 0), maxN = 0, maxEE = 5, truncQ = 4, rm.phix = TRUE, compress = TRUE, multithread = TRUE, verbose = TRUE, and minLen = 50. A rarefaction curve was generated for each sample based on the number of reads for each unique ASV sequence. For each rarefaction curve, the minimum number of unique ASV sequences with a slope of 0.002308 or less was examined, and the number of sequences plus 1 was used as the sampling depth for rarefying. Rarefaction was conducted using the vegan package (ver. 2.5.7) in the R software. Rdp_train_set_18 (https://zenodo.org/record/4310151#.Yg8oWOjP1PY, accessed on 1 January 2022) was used to assign a genus name to each unique ASV sequence.

### 2.8. Statistical Analysis

Continuous data are presented as mean ± standard deviation (SD), while categorical data are presented as frequencies and percentages. The Welch’s *t* test and the Wilcoxon rank-sum test were used for between-group data comparisons, depending on the distribution of the data. Within-group comparisons were conducted using the Friedman test, which was performed on a total of three observations. Observations that were significantly different in the Friedman test were further analyzed using paired *t* test or Wilcoxon signed-rank test, depending on the distribution of the data. *p* values were corrected for multiple testing using the Benjamini–Hochberg method. Welch’s *t* test and paired *t* test were performed using the t test function with paired = FALSE and paired = TRUE. Benjamini–Hochberg multiple test correction was performed using the p.adjust function with method = BH. Friedman test was performed using the friedman.test function.

Alpha diversity of the intestinal microbiota was assessed using the Simpson diversity index.

PerMANOVA analysis was performed to compare the structural similarity of intestinal microbiota. Adonis function in the vegan package (ver. 2.5.7) was used for PerMANOVA analysis, using 9999 permutations. PerMANOVA variance tests were performed using the betadisper function in the vegan package and ANOVA function in the stats package.

Genus-level comparisons of intestinal microbiota were performed using the ALDEx2 package ver. 1.26.0 (https://github.com/ggloor/ALDEx_bioc, accessed on 27 October 2021) in R software ver. 4.1.0. For this comparison, the microbiota abundance count data were subjected to a centered log-ratio (CLR) transformation using the ALDEx2 aldex.clr function, with mc.samples = 128 and denom = “all” as arguments. For between-group comparisons, the Wilcoxon rank sum test was performed using the ALDEx2 aldex.ttest function. For within-group comparisons, the Friedman test was performed using the friedman.test function, and the Wilcoxon signed rank test was performed using the aldex.ttest function in ALDEx2. Correction for multiple testing was conducted using the Benjamini–Hochberg method, using the p.adjust function and method = BH.

The threshold for statistical significance was set at a *p* value of 0.05.

### 2.9. Large Japanese Gut Microbiota Database

The large Japanese gut microbiota database used for the analysis was SymMAD (Symbiosis Microbiome Analysis Database, not available to the public due to privacy reasons), which contains 16S rRNA metagenomic data. This database contains two major datasets. The first is a dataset of intestinal bacterial DNA extracted from stool samples collected from a Japanese population by the former Benno Laboratory, RIKEN Baton Zone Program, RIKEN Cluster for Science, Technology and Innovation Hub (Saitama, Japan) and analyzed by the Japan Agricultural Frontier Development Organization (Tokyo, Japan), which is licensed by Symbiosis Solutions Inc. The second dataset was generated by Symbiosis Solutions Inc., which obtained consent for individual participation in the study. The two datasets have a combined total of 23,139 samples (as of March 2022). SymMAD contains data on intestinal microbiota as well as information from questionnaires on the participants’ disease status and lifestyle, including eating habits.

## 3. Results

### 3.1. Selection of Analysis Population

**Figure 1:**
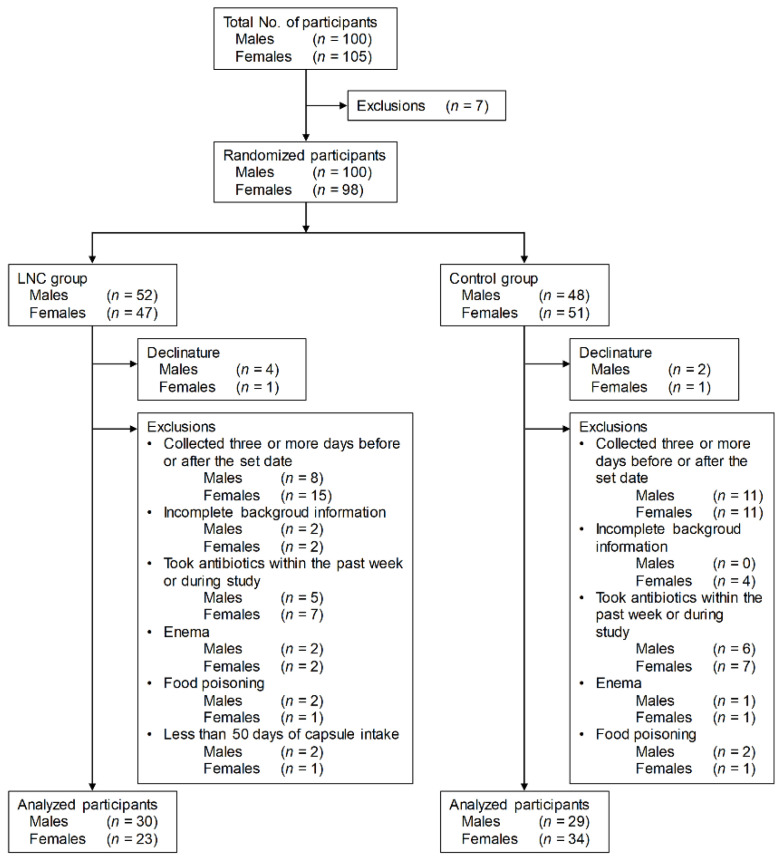
Screening of participants for inclusion in the study. LNC, lyophilized natto capsules.

Males and females aged 20 to 89 years living in Kohoku, Saga Prefecture, Japan, were recruited into the study based on the screening criteria (Figure 1). Participants who met the exclusion criteria were excluded from the 205 townspeople monitors (100 males and 105 females) who gave consent to participate in the study. Participants were randomly divided into two groups. Participants who withdrew during the study, whose stool samples were collected three or more days before or after the set date, who did not respond to the questionnaire, who used antibiotics in the week before and during the study period, who had their stool collected by enema, and who suffered from food poisoning during the study period were also excluded. Also excluded from the LNC group were participants who ingested the test food for less than 50 days.

The final analysis included 30 males and 23 females in the LNC group and 29 males and 34 females in the control group.

There were no significant differences in age, weight, or BMI between participants of either sex in the LNC and control groups (Table 1). There were also no significant differences in the intake of fermented foods, including natto, between participants of either sex in the LNC and control groups.

### 3.2. Variations in Weight, BMI, and Defecation Status

**Figure 2:**
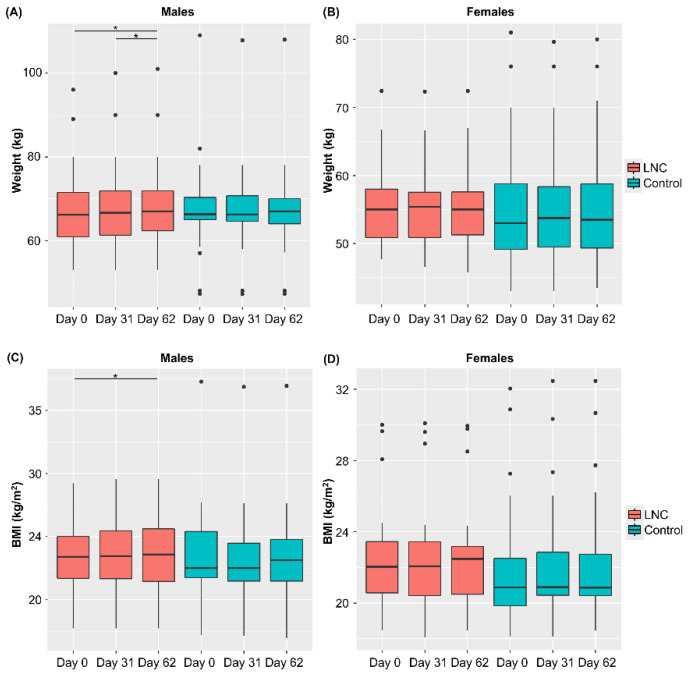
Changes in body weight and BMI following ingestion of SONOMONO NATTO POWDER CAPSULES^TM^. Each dot represents outliers. (**A**) Wight of males, (**B**) weight of females, (**C**) BMI of males, (**D**) BMI of females. Significant increase in weight was observed between samples collected from males in the LNC (lyophilized natto capsules) group on Day 0 and 62 and on Day 31 and 62. Furthermore significant increase in BMI was observed between samples collected from males in the LNC group on Day 0 and 62. * *p* < 0.05.

Changes in weight and BMI during the study period were analyzed (Figure 2). Weight and BMI increased significantly from Day 0 to 62 in males in the LNC group, but not in males in the control group. No significant changes in body weight or BMI were observed in females in the LNC and control groups. There were also no significant changes in the defecation status in individuals of either sex.

### 3.3. Changes in Intestinal Microbiota

We analyzed the intestinal microbiota using 16S rRNA sequence data and generated microbiota composition data from the participants’ stool samples.

We used this intestinal microbiota composition data to analyze the diversity of each sample (α-diversity: Simpson index) and the distance of the intestinal microbiota between samples (β-diversity). There were no significant differences in α-diversity and β-diversity of the intestinal microbiota at the beginning of the study (Day 0) between males and females in the LNC and control groups (Table S1). Additionally, when genus-level CLR-transformed abundance was compared with the ALDEx2 pipeline, no significant differences in abundance were observed in the bacterial genus in participants of both sexes on Day 0 (Table S2).

**Figure 3:**
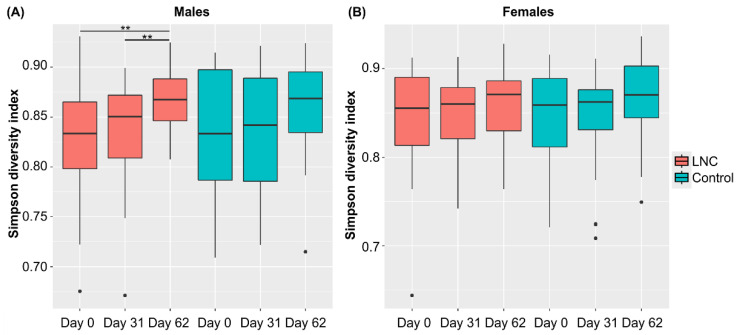
Changes in the diversity of intestinal microbiota following ingestion of SONOMONO NATTO POWDER CAPSULES^TM^. Box plot of α-diversity calculated using the Simpson diversity index. Each dot represents outliers. (**A**) Males, (**B**) females. Significant increase in α-diversity was observed between samples collected from males in the LNC (lyophilized natto capsules) group on Day 0 and 62 and on Day 31 and 62. ** *p* < 0.01.

Analysis of changes in α-diversity during the study period revealed that α-diversity in males in the LNC group was significantly higher on Day 62 than on Day 0 and 31 (*p* < 0.01, Figure 3). No significant changes in α-diversity were observed in males in the LNC group and in females in the LNC and control groups. Significant differences in β-diversity were also detected between males and females in the LNC and control groups (Table S3).

CLR-transformed abundances were compared at the genus level. The increase or decrease in the abundance of each genus was analyzed by calculating the mean relative abundance. Some genera showed significant differences in abundance between participants in the LNC group. The genera whose abundance changed significantly among males in the LNC group were *Bacteroides*, *Bifidobacterium*, *Blautia*, *Collinsella*, *Phocaeicola*, and unclassified bacteria (Table 2A). *Bifidobacterium* abundance differed significantly between males in the LNC and control groups, although the difference in abundance between Day 0 and 62 was significant only among males in the LNC group (Table 2A,B). The abundance of *Bacteroides*, *Collinsella*, *Phocaeicola*, and unclassified bacteria also differed significantly in males in the control group, and the increasing and decreasing trends were consistent between males in LNC and control groups.

*Bifidobacterium*, *Faecalibacterium*, *Parabacteroides*, and unclassified bacteria abundance changed significantly in females in the LNC group (Table 2C). Similar to males, *Bifidobacterium* abundance changed significantly among females in LNC and control groups, although the change between Day 0 and 62 was only significant in females in the LNC group (Table 2C,D). *Faecalibacterium* abundance changed only in females in the LNC group, with significant changes being observed between samples collected on Day 31 and 62. However, no significant differences were observed between samples collected on Day 0 and 62. The relative abundance of *Parabacteroides* in females in the LNC group increased significantly between Day 0 and 31 but decreased significantly between Day 31 and 62. Females in the LNC group had significantly lower *Parabacteroides* abundance on Day 62 than on Day 0. The abundance of unclassified bacteria also changed significantly in females in the control group, and the increasing and decreasing trends were consistent between females in the LNC and control groups.

### 3.4. Characteristics of Male Participants with a Significant Increase in Blautia Abundance in the LNC Group

**Figure 4:**
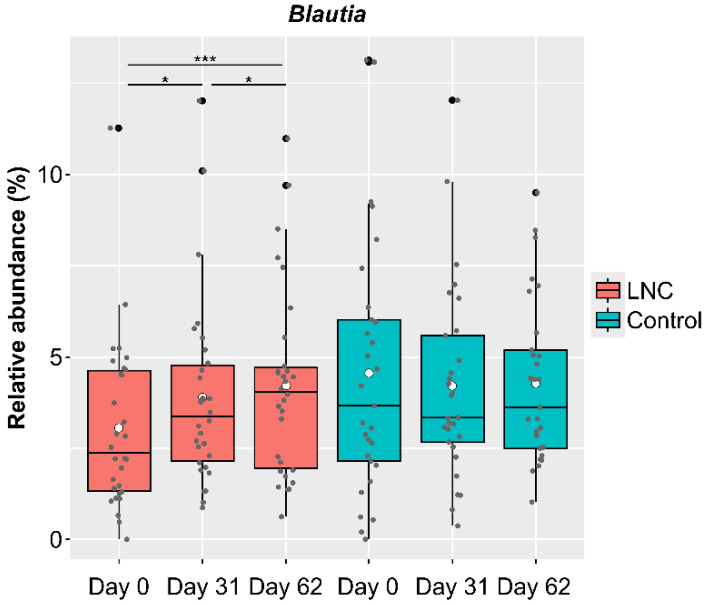
Changes in the relative abundance of *Blautia* among male study participants. Black dots show outliers and gray dots show values for each participant. Comparison of CLR-transformed *Blautia* abundance showed significant differences between Day 0 and 31 and between Day 31 and 62. There were significant increases in *Blautia* abundance between Day 0 and 31, between Day 31 and 62, and between Day 0 and 62 among males in the LNC (lyophilized natto capsules) group. In contrast, no significant changes were observed in males in the control group. * *p* < 0.05; *** *p* < 0.001.

Genus-level analysis showed significant changes in CLR-transformed abundance of *Blautia*, with an increase in relative abundance in males in the LNC group. However, there were a certain number of participants whose *Blautia* abundance had not increased by the end of the study (Day 62) (Figure 4). To clarify the differences between participants whose *Blautia* abundance increased and those whose *Blautia* abundance did not increase, we compared the intestinal microbiota data with the responses to the background survey taken at the beginning of the study (Day 0). We selected participants whose relative *Blautia* abundance was below the mean (3.06%) on Day 0. Of these, participants whose relative *Blautia* abundance increased to the mean (4.22%) or above at the end of the study were defined as the IB group (increased *Blautia* group: *n* = 9), while those whose relative *Blautia* abundance remained below the mean were classified as the NB group (no change in *Blautia* group: *n* = 9).

**Figure 5:**
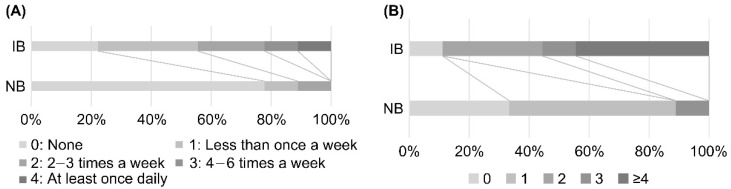
Comparison of dietary habits of individuals in IB (increased *Blautia*) and NB (no change in *Blautia*) groups. (**A**) Comparison of the frequency of natto intake in the previous month. There was a significant difference (*p* < 0.05) between individuals in the IB and NB groups, with those in the IB group consuming natto more frequently than those in the NB group. (**B**) Comparison of the number of fermented foods consumed in the previous month. The following eight categories of fermented foods were considered: pickles, cheese, miso soup, natto, koji food, sake lees food, vinegar, and others. There was a significant difference (*p* < 0.01) between the IB and NB groups, with more fermented foods consumed in the IB group than in the NB group.

Comparison of the background survey responses of individuals in the IB and NB groups showed a significant difference in the frequency of natto intake in the previous month, with individuals in the IB group consuming natto more frequently than individuals in the NB group (*p* < 0.05, Figure 5A). Individuals in the IB group also consumed significantly more fermented foods (fermented pickles, cheese, miso soup, natto, koji food, sake lees food, vinegar, and others) in the previous month compared with individuals in the NB group (*p* < 0.01, Figure 5B). Analysis of the CLR-transformed abundance of each genus on Day 0 showed a significant difference in *Bifidobacterium* only, with higher relative abundance of *Bifidobacterium* in the IB group than in the NB group (Table S4).

**Figure 6:**
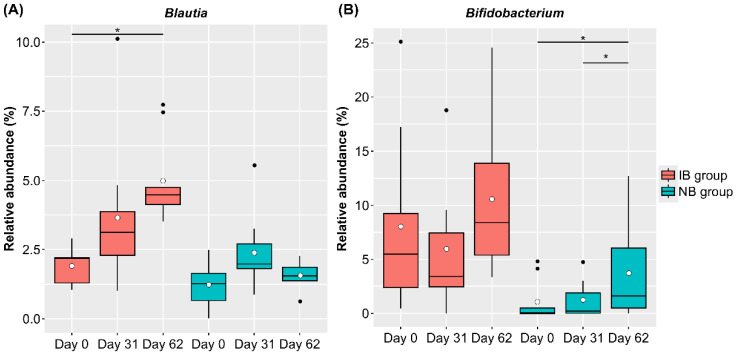
Relative abundance of bacterial genera that changed significantly in the IB (increased *Blautia*) and NB (no change in *Blautia*) groups. Each dot represents outliers. (**A**) *Blautia*, (**B**) *Bifidobacterium*. Comparison of CLR-transformed abundance of each genus using the ALDEx2 pipeline showed that *Blautia* abundance in the IB group was significantly higher on Day 62 than on Day 0, while *Bifidobacterium* abundance in the NB group was significantly higher on Day 62 than on Day 0 and on Day 31. * *p* < 0.05.

We also compared changes in the intestinal microbiota due to the intake of SONOMONO NATTO POWDER CAPSULES^TM^. Analysis of changes in CLR-transformed abundance at the genus level in each group showed a significant increase in *Blautia* in the IB group and a significant increase in *Bifidobacterium* in the NB group (Figure 6).

There were no significant differences in defecation status between individual in the IB and NB groups. In addition, there was no significant difference in CLR-transformed abundance of *Bifidobacterium* between those who were in the habit of consuming fermented foods and those who were not in the habit.

### 3.5. Baseline-Specific Analysis of Bifidobacterium in Females in the LNC Group

**Figure 7:**
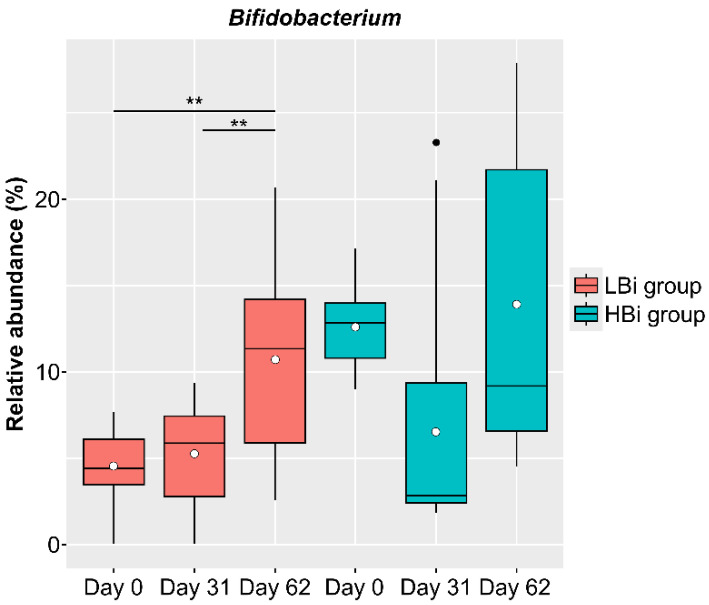
Changes in relative abundance of *Bifidobacterium* in participants in LBi (low *Bifidobacterium*) and HBi (high *Bifidobacterium*) groups. Each dot represents outliers. Comparison of the abundance of each genus in the ALDEx2 pipeline showed a significant increase in abundance between Day 0 and 62 and between Day 31 and 62 in the LBi group. ** *p* < 0.01.

The baseline condition of the intestinal microbiota of females in the LNC group may also affect the changes observed in the intestinal microbiota following the ingestion of SONOMONO NATTO POWDER CAPSULES^TM^. Therefore, we compared the effects of ingesting SONOMONO NATTO POWDER CAPSULES^TM^ on the intestinal microbiota by dividing the LNC group into two: one with high *Bifidobacterium* abundance on Day 0 and the other with low *Bifidobacterium* abundance on Day 0. The LBi (Low *Bifidobacterium*: *n* = 14) group included females whose relative *Bifidobacterium* abundance on Day 0 was below average, while the HBi (High *Bifidobacterium*: *n* = 9) group included females whose relative *Bifidobacterium* abundance was above average on Day 0. The results showed a significant increase in *Bifidobacterium* in the LBi group (Figure 7), but no significant change in the HBi group. Further analysis found no differences in the frequency of consumption of fermented foods between participants in the LBi and Hbi groups.

### 3.6. Effects of Ingesting SONOMONO NATTO POWDER CAPSULES^TM^ Based on Analysis of the SymMAD Database

**Figure 8:**
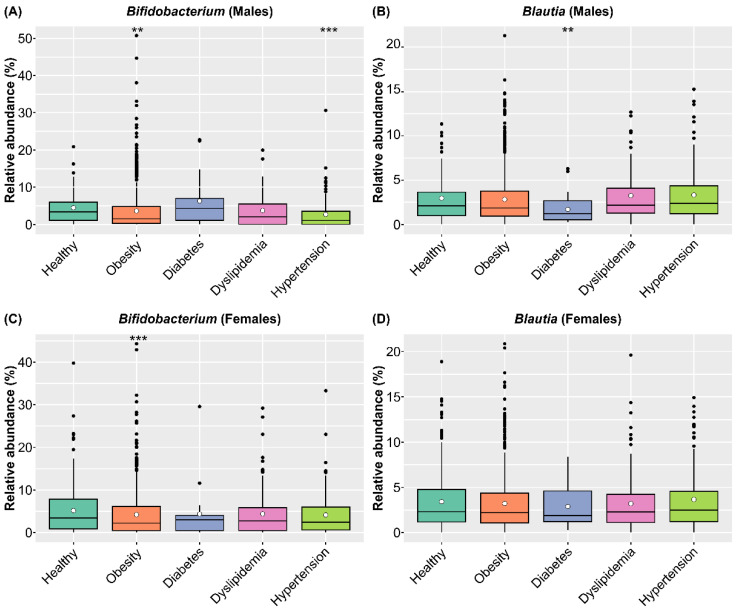
Relative abundance of *Bifidobacterium* and *Blautia* in healthy participants and participants with different diseases in the SymMAD database. (**A**) Relative abundance of *Bifidobacterium* in males. (**B**) Relative abundance of *Blautia* in males. (**C**) Relative abundance of *Bifidobacterium* in females. (**D**) Relative abundance of *Blautia* in females. Each dot represents an outlier. The abundance of each genus in healthy and sick participants was compared using the ALDEx2 pipeline. Obese participants of both sexes had significantly lower abundance of *Bifidobacterium* compared with healthy participants. In males, participants with hypertension had significantly lower abundance of *Bifidobacterium* compared with healthy participants, while participants with diabetes had significantly lower levels of *Blautia* compared with healthy participants. ** *p* < 0.01 and *** *p* < 0.001.

To clarify the medical effects of consuming SONOMONO NATTO POWDER CAPSULES^TM^, we compared the abundance of *Bifidobacterium* and *Blautia* in healthy and diseased participants in the SymMAD database. We included participants aged 40–79 years old with obesity (BMI ≥ 25), diabetes, dyslipidemia, and hypertension, which are major lifestyle-related diseases (Table S5). Comparison of intestinal microbiota of healthy and sick participants using the ALDEx2 pipeline showed that obese participants of both sexes had significantly lower abundance of *Bifidobacterium* compared with healthy participants. In males, individuals with hypertension had a significantly lower abundance of *Bifidobacterium* compared with healthy participants, while individuals with diabetes had significantly lower levels of *Blautia* compared with healthy participants (Figure 8 and Table S6).

## 4. Discussion

Males who ingested SONOMONO NATTO POWDER CAPSULES^TM^ had a significant increase in body weight and BMI. However, the changes were within an appropriate range and did not affect the health status of the participants.

The α-diversity (Simpson diversity index) of the genus of the intestinal microbiota was significantly higher only in males in the LNC group, suggesting that SONOMONO NATTO POWDER CAPSULES^TM^ may increase α-diversity in males. Analysis of β-diversity showed significant differences between both sexes in the LNC group. However, significant differences were also detected in the control group, and it is therefore not clear whether the change in β-diversity can be attributed to the SONOMONO NATTO POWDER CAPSULES^TM^.

Analysis of changes in the genera of the intestinal microbiota revealed that *Bacteroides*, *Bifidobacterium*, *Blautia*, *Collinsella*, *Phocaeicola*, and an unclassified genus were significantly altered in males in the LNC group, while *Bifidobacterium*, *Faecalibacterium*, *Parabacteroides*, and unclassified bacteria were significantly altered in females in the LNC group. Among these genera, we considered the genus that showed a significant difference in the only LNC group between Day 0 and 62 as the genus affected by ingestion. The genera whose abundances may have changed as a result of ingesting SONOMONO NATTO POWDER CAPSULES^TM^ were *Blautia* and *Bifidobacterium* in males and *Bifidobacterium* in females, both of which showed an increase in relative abundance. Furthermore, *Bacteroides*, *Collinsella*, *Phocaeicola*, and unclassified bacteria in males and unclassified bacteria in females showed significant changes even in the control group, and the direction of increase or decrease was the same in the LNC and control groups; thus, the changes could not be attributed solely to the intake of SONOMONO NATTO POWDER CAPSULES^TM^. Although there were significant changes in the abundance of *Faecalibacterium* and *Parabacteroides* in females, the changes between Day 0 and 62 were not significant, and we could not conclude from this experiment alone that the changes in abundance were due to ingesting the capsules.

*Bifidobacterium*, which tended to increase in both sexes following the ingestion of SONOMONO NATTO POWDER CAPSULES^TM^, is a bacterium that breaks down sugar and produces acetic and lactic acids. *Bifidobacterium* abundance also increases with natto intake [14]. Soybeans, natto’s raw material, are rich in oligosaccharides such as stachyose and raffinose [19], which have been reported to increase *Bifidobacterium* abundance in human feces [20,21,22]. The ratio and content of soybean oligosaccharides change during the fermentation process of natto production, with stachyose and raffinose abundance decreasing and manninotriose abundance increasing [23,24]. Similar to stachyose and raffinose, manninotriose increases *Bifidobacterium* abundance in human feces [25]. Although the specific mechanism of action is largely unknown, it is believed that the probiotic action of *Bacillus* requires germination of *Bacillus* spores in the intestinal tract and metabolic activity of the cells [13,26]. Takemura et al. reported that *B. subtilis* MC1 spores reached the intestine in a live state following the ingestion of natto [27]. In addition, Hatanaka et al. used in vitro stomach and small intestine and colon models to show that *B. subtilis* C-3102 spores increase *Bifidobacterium* abundance in the stool, with 99% of the C-3102 spores being viable and 8% germinating [28]. Although no change was observed in *Bacillus* abundance in this study, it is possible that *B. subtilis var. natto* SONOMONO reached the intestine in a live state and contributed to the increase in *Bifidobacterium* abundance.

Comparing the dietary habits and intestinal microbiota of males with increased *Blautia* abundance (IB group) and no change in *Blautia* abundance (NB group) in the LNC group showed that individuals in the IB group consumed fermented foods, including natto, significantly more frequently and had significantly higher *Bifidobacterium* abundance at baseline. Relative *Bifidobacterium* abundance increased with the intake of natto [14] and amazake [29] containing koji and sake lees and kimchi [30]. Therefore, differences in the relative abundance of *Bifidobacterium* at baseline between participants in the IB and NB groups could be attributed to the frequency of consuming fermented foods, including natto.

The increase in *Bifidobacterium* abundance following the intake of natto is more pronounced in individuals with low baseline *Bifidobacterium* abundance [27]. Analysis of males in the LNC group whose *Blautia* abundance increased (IB group) and those whose *Blautia* abundance did not increase (NB group), and comparison of females in the LNC group who had high *Bifidobacterium* abundance (HBi group) and those who had low *Bifidobacterium* abundance (LBi group) at baseline revealed that the increase in *Bifidobacterium* due to SONOMONO NATTO POWDER CAPSULES^TM^ intake was more pronounced in individuals with lower relative *Bifidobacterium* abundance at baseline.

*Blautia*, whose abundance was significantly higher in males following the ingestion of SONOMONO NATTO POWDER CAPSULES^TM^, consumes sugars and produces acetic, lactic, and succinic acids as metabolites [31,32,33]. This genus is relatively new, created in 2008 when certain species previously classified as *Clostridium* and *Ruminococcus* were reclassified into this genus [31]. Although omega-3 fatty acids [34], caffeine [35], and RS4-resistant starch [36] increase *Blautia* abundance, these components are not abundant in SONOMONO NATTO POWDER CAPSULES^TM^. Plichta et al. showed that the metabolic activity of *Blautia hydrogenotrophica* was activated in the presence of *Bifidobacterium bifidum*, suggesting the possibility of cross-feeding between *B*. *hydrogenotrophica* and *Bi*. *Bifidum* [37]. Although species-level analysis was not conducted in this study, the cross-feeding between *Bifidobacterium* and *Blautia* may be one of the reasons for the increase in *Blautia* in males with high *Bifidobacterium* abundance. In addition, although this study was conducted over a two-month period, when males with low relative *Bifidobacterium* abundance at baseline continuously ingested SONOMONO NATTO POWDER CAPSULES^TM^, *Bifidobacterium* abundance increased due to the effect of *B. subtilis var. natto* SONOMONO, leading to an increase in *Blautia* abundance due to the cross-feeding effect. Furthermore, there was no increase in *Blautia* abundance in females following ingestion of SONOMONO NATTO POWDER CAPSULES^TM^, indicating that the effects of ingestion differed between males and females. This may be attributed to the existence of sex differences in the intestinal microbiota [38].

*Bifidobacterium* support human health and protect against infection by producing antimicrobial peptides and intestinal pH-lowering acetic and lactic acids [39,40,41,42]. Moreover, they suppress pathogen growth [43], and induce immunostimulatory pathways via other molecular mechanisms [44,45]. *Blautia* is considered a useful bacteria because it is abundant in people with lower visceral fat area [46]. The relative abundance of *Blautia* is also lower in patients with colorectal cancer [47] and diabetes [48]. In addition, comparison of the intestinal microbiota of healthy people in 12 countries, including Japan, showed that Japanese people had the highest relative abundance of *Blautia* and *Bifidobacterium* [11]. Based on these observations, the increase in the relative abundance of *Bifidobacterium* and *Blautia* following the intake of SONOMONO NATTO POWDER CAPSULES^TM^ is likely to improve the dysbiosis associated with decreased *Bifidobacterium* and *Blautia*, suggesting that it is useful as a probiotic product. In particular, it may be useful for improving the intestinal microbiota of people who consume fermented foods (including natto) infrequently in their daily diet and have low *Bifidobacterium* abundance. Furthermore, analysis of data from the large SymMAD database showed that obese participants of both sexes had significantly lower *Bifidobacterium* abundance compared with healthy participants. Among the males, participants with hypertension had significantly lower abundance of *Bifidobacterium* compared with healthy participants, while participants with diabetes had significantly lower levels of *Blautia* compared with healthy participants. This suggests that changes in the intestinal microbiota caused by the ingestion of SONOMONO NATTO POWDER CAPSULES^TM^ may be effective at preventing and treating obesity, hypertension, and diabetes in Japanese populations.

In this study, we observed the changes in relative abundance of the intestinal microbiota by 16S rRNA metagenomic analysis. However, an increase or decrease in relative abundance does not necessarily correspond to an increase or decrease in the actual number of intestinal bacteria. Even if the relative abundance is increasing, the actual number of cells may remain unchanged or even decrease. Unfortunately, this study did not measure the number of cells in each intestinal bacterium and could not determine the actual changes in the number of intestinal bacteria. Future studies measuring not only the relative abundance but also the cell count of intestinal bacteria are needed for more detailed analysis of changes in the intestinal microbiota.

This study revealed that ingesting SONOMONO NATTO POWDER CAPSULES^TM^, which contains approximately 1.8 × 10^10^ CFUs of *B. subtilis var. natto* SONOMONO spores, increased the relative abundance of *Bifidobacterium* and *Blautia* in males and *Bifidobacterium* in females. Using the large-scale SymMAD database, we showed that changes in the intestinal microbiota caused by consuming SONOMONO NATTO POWDER CAPSULES^TM^ may prevent or treat lifestyle-related diseases, including diabetes, whose incidence is on the rise among the residents of Kohoku Town. Additionally, the ability of SONOMONO NATTO POWDER CAPSULES^TM^ to increase the abundance of *Bifidobacterium* and *Blautia* was dependent on the relative baseline abundance of *Bifidobacterium* in both males and females. This suggests that it is important to understand the intestinal microbiota prior to consuming food for the purpose of improving the intestinal microbiota, and to select food based on the contents of the intestinal microbiota.

## Figures and Tables

**Table 1:** Characteristics of the study population at the beginning of the study (Day 0).

	Males			Females		
	LNC	Control	*p* Value	LNC	Control	*p* Value
Number of samples	30	29		23	34	
Age	53.90 ± 14.91	49.52 ± 16.41	0.278	52.48 ± 15.58	53.76 ± 15.74	0.766
Weight (kg)	67.71 ± 9.64	67.93 ± 10.6	0.710	55.63 ± 6.14	55.11 ± 8.81	0.597
BMI (kg/m^2^)	23.18 ± 2.8	23.51 ± 3.58	0.964	22.59 ± 3.06	21.93 ± 3.19	0.229

Values are mean ± SD. BMI, body mass index. LNC, lyophilized natto capsules.

**Table 2:** Changes in the relative abundance of each genus.

(**A**) Bacterial genera with significantly altered abundance in males in the LNC group							
**Genus**	**Relative Abundance (%)**			***p* Value (Friedman Test)**	***p* Value (Wilcoxon Signed Rank Test)**		
	**Day 0**	**Day 31**	**Day 62**		**Day 0 vs. 31**	**Day 31 vs. 62**	**Day 0 vs. 62**
*Bacteroides*	5.63 ± 5.92	6.83 ± 6.48	4.57 ± 4.41	0.012	0.031	0.304	0.304
*Bifidobacterium*	6.17 ±8.20	3.65 ± 4.17	7.23 ± 5.81	0.000	0.510	0.000	0.000
*Blautia*	3.06 ± 2.32	3.90 ± 2.56	4.22 ± 2.60	0.001	0.029	0.047	0.000
*Collinsella*	2.34 ± 3.79	0.63 ± 1.01	3.19 ± 2.59	0.001	0.345	0.002	0.009
*Phocaeicola*	20.96 ± 16.41	22.95 ± 14.59	15.59 ± 12.53	0.012	0.016	0.103	0.331
Unclassified	10.65 ± 7.25	10.63 ± 4.43	12.92 ± 7.52	0.000	0.140	0.006	0.000
(**B**) Changes in relative abundance of genera shown in (**A**) in males in the control group							
**Genus**	**Relative abundance (%)**			***p* value (Friedman test)**	***p* value (Wilcoxon signed rank test)**		
	**Day 0**	**Day 31**	**Day 62**		**Day 0 vs. 31**	**Day 31 vs. 62**	**Day 0 vs. 62**
*Bacteroides*	5.73 ± 5.62	7.59 ± 5.69	5.12 ± 4.05	0.004	0.058	0.340	0.095
*Bifidobacterium*	8.51 ± 7.85	4.78 ± 3.54	9.45 ± 8.44	0.000	0.181	0.001	0.098
*Blautia*	4.56 ± 3.51	4.20 ± 2.65	4.28 ± 2.23	0.073	-	-	-
*Collinsella*	1.93 ± 2.75	0.34 ± 0.85	2.97 ± 2.17	0.000	0.119	0.000	0.004
*Phocaeicola*	24.52 ± 14.36	28.75 ± 13.87	21.08 ± 13.80	0.000	0.022	0.186	0.396
Unclassified	9.15 ± 4.48	10.79 ± 5.02	10.96 ± 5.54	0.000	0.067	0.067	0.003
(**C**) Bacterial genera with significantly altered abundance in females in the LNC group							
**Genus**	**Relative abundance (%)**			***p* value (Friedman test)**	***p* value (Wilcoxon signed rank test)**		
	**Day 0**	**Day 31**	**Day 62**		**Day 0 vs. 31**	**Day 31 vs. 62**	**Day 0 vs. 62**
*Bifidobacterium*	7.70 ± 4.68	5.77 ± 5.15	11.99 ± 7.14	0.001	0.448	0.000	0.003
*Faecalibacterium*	6.94 ± 4.67	5.30 ± 2.96	8.42 ± 6.27	0.002	0.710	0.011	0.066
*Parabacteroides*	3.05 ± 2.63	4.47 ± 3.36	2.37 ± 1.77	0.008	0.004	0.050	0.424
Unclassified	13.39 ± 8.98	15.84 ± 8.72	16.00 ± 9.38	0.001	0.018	0.254	0.001
(**D**) Changes in relative abundance of genera shown in (**C**) in females in the control group							
**Genus**	**Relative abundance (%)**			***p* value (Friedman test)**	***p* value (Wilcoxon signed rank test)**		
	**Day 0**	**Day 31**	**Day 62**		**Day 0 vs. 31**	**Day 31 vs. 62**	**Day 0 vs. 62**
*Bifidobacterium*	9.77 ± 7.32	6.54 ± 4.76	9.94 ± 6.14	0.020	0.224	0.019	0.265
*Faecalibacterium*	5.79 ± 4.74	4.84 ± 4.00	5.40 ± 3.92	0.152	-	-	-
*Parabacteroides*	3.53 ± 4.89	4.21 ± 4.73	3.63 ± 5.14	0.282	-	-	-
Unclassified	10.83 ± 7.90	11.03 ± 7.65	13.91 ± 10.91	0.000	0.301	0.001	0.001

Values are mean ±SD.

## Data Availability

The data presented in this study are available on request from the corresponding author. The data are not publicly available due to privacy reasons.

## Supplementary Materials

The following supporting information can be downloaded at: https://www.mdpi.com/article/10.3390/nu14183839/s1, Table S1: Comparison of diversity (genus level) on Day 0; Table S2: Comparison of genus-level intestinal microbiota on Day 0; Table S3: Changes in β-diversity at the genus level; Table S4: Genera whose abundance differed significantly between IB and NB groups. Table S5: Sample size for each group; Table S6: Comparison of genus-level intestinal microbiota in each disease and healthy group.
