# Supplementary material for: Fluctuations in Intestinal Microbiota Following Ingestion of Natto Powder Containing Bacillus subtilis var. natto SONOMONO Spores: Considerations Using a Large-Scale Intestinal Microflora Database
Source: Nutrients. 2022 Sep 16;14(18):3839. doi: 10.3390/nu14183839 (PMC9505718; doi:10.3390/nu14183839)
Supplement: Supplementary file 1 [file nutrients-14-03839-s001.zip › nutrients-1884559-supplementary.pdf]

Supplementary Tables

Table S1. Comparison of diversity (genus level) on Day 0.

(A) α-diversity on Day 0			
**Gender**	**Group**	**Simpson index**	**p-value**
Males	LNC	0.827±0.053	0.316
	Control	0.842±0.056	
Females	LNC	0.848±0.059	0.705
	Control	0.846±0.054	

(B) β-diversity on Day 0		
**Gender**	**p-value**	
	**PerMANOVA**	**dispersion**
Males	0.376	0.155
Females	0.287	0.038

Values are mean ± SD.

Table S2. Comparison of genus-level intestinal microbiota on Day 0.

(A) Comparison of genus-level intestinal microbiota in males on Day 0			
**Genus**	**Relative abundance (%)**		**p-value**
	**LNC**	**Control**	
Phocaeicola†	20.96	24.52	0.233
Prevotella	11.67	6.23	0.299
Unclassified	10.65	9.15	0.701
Bifidobacterium	6.17	8.51	0.054
Bacteroides	5.63	5.73	0.663
Faecalibacterium	5.08	4.79	0.714
Megamonas	4.95	2.98	0.336
Blautia	3.06	4.56	0.098
Fusicatenibacter	2.59	3.08	0.551
Collinsella	2.34	1.93	0.765
Parabacteroides	2.33	2.88	0.438
Streptococcus	2.32	1.32	0.449
Agathobacter	1.99	2.31	0.811
Ruminococcus	1.71	1.60	0.592
Alistipes	1.57	1.41	0.830
Holdemanella	1.54	1.08	0.663
Faecalibacillus	1.48	1.29	0.749
Dorea	1.22	1.58	0.405
Phascolarctobacterium	1.21	0.87	0.519
Lachnospira	1.09	0.85	0.631

(B) Comparison of genus-level intestinal microbiota in females on Day 0			
Genus	Relative abundance (%)		p-value
	LNC	Control	
Phocaeicola†	23.93	17.31	0.092
Unclassified	13.39	10.83	0.327
Bacteroides	9.76	11.11	0.835
Bifidobacterium	7.70	9.77	0.429
Faecalibacterium	6.94	5.79	0.313
Fusicatenibacter	3.82	2.55	0.143
Blautia	3.18	3.92	0.369
Parabacteroides	3.05	3.53	0.585
Alistipes	2.88	4.11	0.501
Agathobacter	2.54	2.02	0.684
Prevotella	2.30	6.99	0.477
Ruminococcus	2.23	2.36	0.787
Anaerostipes	1.38	0.83	0.149
Megamonas	1.14	0.44	0.457
Holdemanella	1.11	1.38	0.396
Lachnospira	1.10	1.37	0.584
Dorea	1.01	0.87	0.648
Streptococcus	0.99	2.24	0.286
Mediterraneibacter	0.84	0.47	0.103
Collinsella	0.83	1.17	0.705

The list includes the 20 most abundant genera identified in the LNC group.

† Phocaeicola is a genus reclassified from Bacteroides in 2019.

Table S3. Changes in β-diversity at the genus level.

Gender	Group	Comparison		p-value	
				**PerMANOVA**	**dispersion**
Males	LNC	Day 0	Day 31	0.584	0.046
		Day 31	Day 62	0.010	0.046
		Day 0	Day 62	0.010	0.763
	Control	Day 0	Day 31	0.396	0.046
		Day 31	Day 62	0.010	0.084
		Day 0	Day 62	0.010	0.606
Females	LNC	Day 0	Day 31	0.842	0.333
		Day 31	Day 62	0.010	0.118
		Day 0	Day 62	0.010	0.525
	Control	Day 0	Day 31	0.545	0.286
		Day 31	Day 62	0.010	0.392
		Day 0	Day 62	0.010	0.797

Table S4. Genera whose abundance differed significantly between IB and NB groups.

Genus	Relative abundance (%)						p-value
	IB			NB			
Bifidobacterium	8.02	±	8.19	1.08	±	1.93	0.003

Values are mean ±SD.

Table S5. Sample size for each group.

	**Males**	**Females**
Healthy	52	240
Obesity	680	663
Diabetes	30	17
Dyslipidemia	72	137
Hypertension	112	108

Table S6. Comparison of genus-level intestinal microbiota in each disease and healthy group.

(A) Comparison of genus-level intestinal microbiota in males

	Healthy	Obesity		Diabetes		Dyslipidemia		Hypertension	
	**RA (%)**	**RA (%)**	**p-value**	**RA (%)**	**p-value**	**RA (%)**	**p-value**	**RA (%)**	**p-value**
Phocaeicola	15.89	19.08	0.373	15.73	0.384	21.60	0.044	17.62	0.790
Unclassified	14.63	12.78	0.053	15.50	0.238	12.47	0.139	14.79	0.810
Bacteroides	10.99	9.70	0.034	13.16	0.812	13.02	0.646	11.40	0.618
Prevotella	10.86	10.75	0.706	3.71	0.523	6.65	0.466	9.07	0.752
Faecalibacterium	8.03	6.26	0.003	6.21	0.007	6.36	0.026	6.50	0.025
Bifidobacterium	4.55	3.70	0.003	6.29	0.831	3.78	0.092	2.75	0.000
Alistipes	3.03	2.41	0.002	4.85	0.372	2.65	0.170	3.28	0.177
Blautia	2.96	2.84	0.558	1.71	0.019	3.28	0.472	3.32	0.577
Agathobacter	2.40	2.00	0.240	0.94	0.006	1.43	0.028	1.77	0.167
Anaerostipes	2.26	1.39	0.005	0.77	0.001	1.58	0.177	1.33	0.030

(B) Comparison of genus-level intestinal microbiota in females

	Healthy	Obesity		Diabetes		Dyslipidemia		Hypertension	
	**RA (%)**	**RA (%)**	**p-value**	**RA (%)**	**p-value**	**RA (%)**	**p-value**	**RA (%)**	**p-value**
Unclassified	17.04	15.28	0.027	18.00	0.792	16.32	0.318	16.63	0.643
Phocaeicola	17.01	16.88	0.764	18.64	0.476	15.45	0.111	17.72	0.723
Bacteroides	13.54	14.40	0.418	10.79	0.177	15.66	0.075	13.68	0.776
Faecalibacterium	7.65	6.98	0.033	6.35	0.658	7.93	0.879	7.29	0.402
Bifidobacterium	5.22	4.26	0.003	4.39	0.323	4.41	0.093	4.15	0.117
Alistipes	4.39	3.50	0.002	2.40	0.036	4.21	0.613	4.38	0.630
Prevotella	4.16	5.48	0.230	7.62	0.269	5.21	0.603	4.59	0.655
Blautia	3.43	3.25	0.351	2.89	0.696	3.21	0.575	3.69	0.693
Parabacteroides	2.42	2.98	0.154	2.28	0.693	2.43	0.757	2.77	0.289
Anaerostipes	2.06	1.61	0.002	1.71	0.551	1.72	0.117	1.57	0.029

Values are mean. RA, relative abundance. The list includes the 10 most abundant genera identified in the healthy group
